# Vesicocolic fistula in myasthenia gravis patient: A case report

**DOI:** 10.1097/MD.0000000000045586

**Published:** 2025-10-31

**Authors:** Xiaohui Liu, Jiao Meng, Baosu Huang, Peng Jin

**Affiliations:** a General Surgery Department, Affiliated Hospital of Shandong Second Medical University, School of Clinical Medicine, Shandong Second Medical University, Weifang, Shandong Province, China.

**Keywords:** case report, colon, intestinal fistula, myasthenia gravis, vesical fistula

## Abstract

**Rationale:**

Vesicocolic fistula is a rare disease, and surgical intervention is the only available treatment, while complications can arise when patients also suffer from myasthenia gravis, a neuromuscular disease that can be exacerbated by the stress of surgery.

**Patient concerns:**

We report a 60-year-old male with a history of heart disease, myasthenia gravis, and other comorbidities who was admitted with complaints of fecaluria for the past 18 months.

**Diagnoses:**

Computed tomography showed bladder cancer involving the distal descending colon, with a high suspicion of a vesicocolic fistula. In addition, multiple diverticula were observed in both the descending and sigmoid colon.

**Interventions:**

In this case, a multidisciplinary approach and careful perioperative management enabled the successful resolution of the fistula while minimizing risks associated with the patient’s underlying condition. Finally, the fistula was successfully solved by laparoscopic surgery.

**Outcomes:**

At 3 months postoperatively, the patient recovered well and did not reappear with the previous symptom.

**Lessons:**

Vesicocolic fistula complicated by myasthenia gravis poses unique challenges in surgical management. We summarized the clinical features and treatment methods of this vesicocolic fistula, hoping to contribute to the development of the surgical treatment strategies for vesicocolic fistula and provide insights for others experiencing similar conditions.

## 1. Introduction

**Figure 1.**
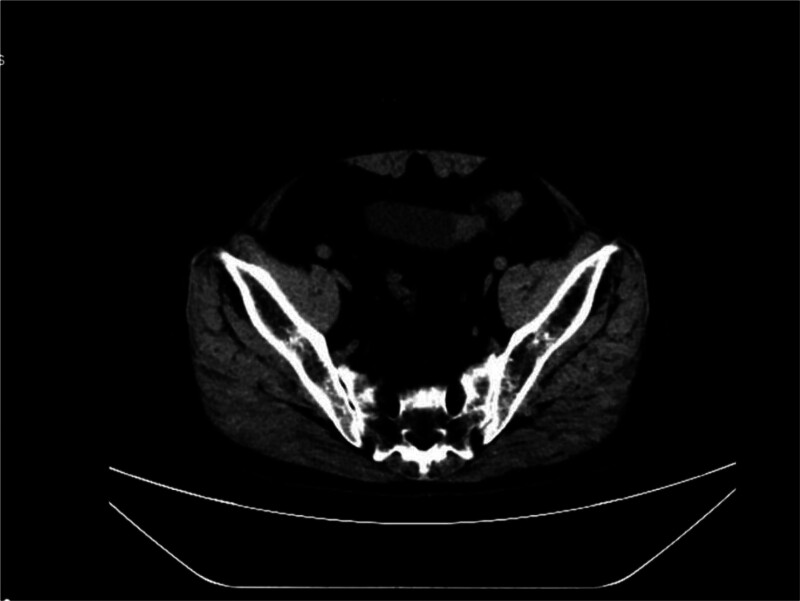
The left posterior wall of the bladder shows localized thickening, with an irregular nodule-like protrusion measuring approximately 2.2 × 1.7 cm, which is adherent to the distal segment of the descending colon.

A 60-year-old male with a history of heart disease, myasthenia gravis, and other comorbidities was admitted with complaints of fecaluria for the past 18 months. The patient is on long-term oral treatment, taking over 20 medications, including methylprednisolone and pyridostigmine bromide. The computed tomography examination (Fig. 1) showed bladder cancer involving the distal descending colon, with a high suspicion of a vesicocolic fistula. Additionally, multiple diverticula were observed in both the descending and sigmoid colon.

A vesicoenteric fistula (VCF) refers to an abnormal connection between the bladder and the intestines, leading to the leakage of urine and intestinal contents. VCFs are rarely reported clinically, both domestically and internationally. Based on their location, they can be classified into vesicoileal fistulas, vesicocolic fistulas, and vesicorectal fistulas.^[1]^ Most vesicocolic fistulas are caused by intestinal diseases, including diverticulitis, intestinal tumors, and Crohn disease. Due to the high prevalence of intestinal diverticulitis in Europe and the United States, approximately 2% of cases of diverticulitis may be complicated by VCFs.^[2]^ In China, intestinal malignancies are the most common, while the incidence of diverticulitis is relatively low, and VCFs caused by diverticulitis are extremely rare.^[3,4]^ It can only be cured through surgical intervention, which typically requires the removal of portions of both the colon and the bladder.^[5]^ The patient’s myasthenia gravis may be aggravated by the stress of surgery, potentially involving the entire muscular system. This can lead to paralysis, respiratory failure, and even death. In addition, surgical procedures requiring organ removal pose significant risks, and the occurrence of postoperative complications, such as an intestinal fistula, can be life-threatening.^[6]^ These factors make the surgery particularly challenging and risky.

## 2. Case report

### 2.1. Patient information

He reported that around 18 months ago, without any obvious cause, he began passing small amounts of urine containing what appeared to be fecal matter. The latter part of the urine had fecal-like particles, but there was no interruption in urination or any blood in the urine. His symptoms became progressively worsened over the past 6 months, prompting him to seek a diagnosis and treatment at our hospital.

The patient had a long-standing history of heart disease, myasthenia gravis, peripheral neuritis, and cerebral infarction. He was on long-term oral treatment, taking over 20 medications, including methylprednisolone and pyridostigmine bromide. No significant positive signs were found on physical examination.

### 2.2. Diagnostic assessment

Computed tomography scan of the chest, upper abdomen, lower abdomen, and pelvis demonstrated: bladder carcinoma involving the distal descending colon, with high suspicion of vesicocolonic fistula formation. Multiple hepatic cysts. Multiple diverticula in the descending and sigmoid colon. Bilateral pulmonary micronodules; clinical correlation and follow-up recommended. Bilateral pulmonary fibrotic foci. Coronary artery calcified plaques. Healed fractures of bilateral 3rd, 4th, and 5th ribs.

### 2.3. Therapeutic intervention

**Figure 2.**
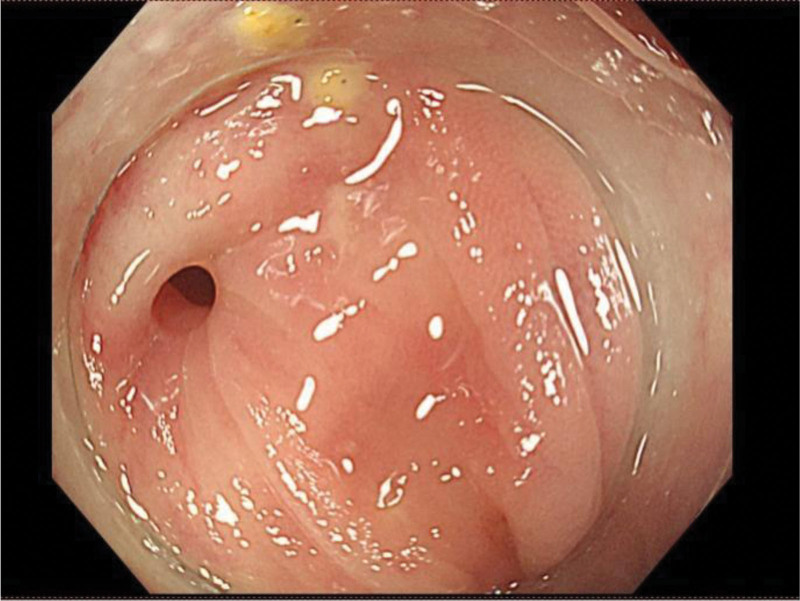
A round opening approximately 4mm in diameter was observed 30 cm from the anal verge, with a smooth surface.

In managing this complex condition, our department has actively prepared and made a rigorous treatment plan for the patients. First, a collaborative approach with the urology department was initiated to perform a colonoscopy and cystoscopy for further evaluation. During the colonoscopy (Fig. 2), a round opening approximately 4 mm in diameter was observed 30 cm from the anal verge, with a smooth surface, but its interior could not be visualized. A subsequent cystoscopy was performed, and a biopsy was taken for pathological examination to rule out tumor-related lesions. After the location of the fistula was precisely identified, our expert team proceeded with a double-mirror combined surgical treatment for the patient. Heavy adhesions around the fistula, caused by inflammation, were encountered during laparoscopy. Part of the colon wall was resected after laparoscopic adhesiolysis, minimizing trauma and preventing intestinal stenosis. Additionally, a portion of the bladder was excised, and the fistula was removed entirely. A colonoscopy was performed during the procedure to assess the closure of the stump, which was found to be successful, with no signs of stenosis. The pathological examination revealed acute and chronic inflammation of the intestinal mucosa, accompanied by erosion. There was significant infiltration of both acute and chronic inflammatory cells, along with localized necrosis, abscess formation, and granuloma formation in the submucosal and muscular layers. The patient made a full recovery and was discharged from the hospital.

### 2.4. Follow-up and outcomes

At 3 months postoperatively, the patient recovered well and did not reappear with the previous symptom.

### 2.5. Informed consent

We obtained informed consent from the patient to publish details of their medical cases and any accompanying images. This study has been approved by the Medical Ethics Committee of Affiliated Hospital of Shandong Second Medical University.

## 3. Discussion

Most vesicocolic fistulas are caused by intestinal diseases, including diverticulitis, intestinal tumors, and Crohn disease. The imaging examination in this case revealed multiple diverticula in the colon, and we suspect that the development of the vesicocolic fistula is most likely related to long-term use of over 20 medications, including hormones.^[7,8]^

Vesicocolic fistulas can lead to several complications, including recurrent urinary tract infections, pelvic abscesses, intestinal obstruction, and peritonitis. Surgical intervention is often required, with the choice of primary or secondary repair depending on the location of the fistula and the patient’s overall health.^[9]^ Nonsurgical treatment is appropriate only for selected patients and typically involves intravenous total parenteral nutrition, intestinal rest, and antibiotics. Therefore, surgical closure of the fistula is essential. The objective of surgical treatment is to isolate and close the affected organ. However, given that the patient in this case has myasthenia gravis, surgery carries the risk of exacerbating the condition, potentially leading to paralysis and respiratory failure in severe cases due to the stress of the procedure. Consequently, repairing fistula repair presents significant challenges. After thorough preoperative evaluation and perioperative preparation, a combined double-mirror operation was performed to treat the patient. Following laparoscopic adhesiolysis, part of the colon wall was resected, which minimized trauma and helped prevent intestinal stenosis. The patient recovered well and was discharged from the hospital shortly after.

Common clinical manifestations of VCFs include abdominal pain, fecaluria, pneumaturia, hematuria, fever, and recurrent urinary tract infections. The possibility of this condition should be considered in cases of refractory urinary tract infections and hematuria. Once a vesicocolic fistula is diagnosed, surgical repair is the preferred treatment.

## Author contributions

**Conceptualization:** Jiao Meng.

**Data curation:** Baosu Huang.

**Writing – original draft:** Xiaohui Liu.

**Writing – review & editing:** Peng Jin.
